# Cervical pregnancy treated with methotrexate combined with traditional Chinese medicine: a case report and literature review

**DOI:** 10.3389/fmed.2025.1723052

**Published:** 2025-12-05

**Authors:** Chuman Zhang, Ying Yang, La Ta

**Affiliations:** 1 Department of Obstetrics and Gynecology, Dandong Central Hospital, Dandong, China; 2 Department of Obstetrics and Gynecology, Dalian Medical University, Dalian, China

**Keywords:** cervical pregnancy, methotrexate (MTX), traditional Chinese medicine, case report, literature review

## Abstract

**Background:**

Cervical pregnancy is a rare form of ectopic pregnancy in which the embryo implants within the cervical canal. With the growing use of assisted reproductive technologies (ARTs), its incidence has gradually increased. Traditionally, management often necessitated hysterectomy due to the high risk of severe hemorrhage. However, conservative approaches using methotrexate (MTX), either alone or in combination with adjunctive therapies, have demonstrated success in preserving fertility.

**Case presentation:**

We present the case of a 38-year-old woman who presented with amenorrhea and prolonged vaginal bleeding. Ultrasonography and elevated serum beta-human chorionic gonadotropin (β-hCG) levels confirmed the diagnosis of cervical pregnancy. The patient was treated with systemic MTX (50 mg/m^2^) in combination with a traditional Chinese medicine (TCM) formula designed to promote blood circulation and resolve stasis. During the treatment, she experienced transient heavy vaginal bleeding that required transfusion but did not necessitate surgical intervention. β-hCG levels declined progressively, ultrasonography confirmed complete resolution of the gestational sac, and menstruation resumed 18 days after treatment completion.

**Conclusion:**

Combined therapy with MTX and TCM may provide an effective, fertility-preserving option for carefully selected patients with cervical pregnancy. TCM may enhance the efficacy of MTX, facilitate tissue resorption, and reduce drug toxicity. Further research is warranted to standardize herbal dosages and clarify the underlying pharmacological mechanisms.

## Introduction

Cervical pregnancy is a rare type of ectopic pregnancy in which the fertilized ovum implants and develops within the cervical canal, with an estimated incidence of approximately one in 8,600 to 12,400 pregnancies. In recent years, the widespread use of assisted reproductive technologies (ARTs) has been associated with an increased incidence of cervical pregnancy. In a study of 91,067 ART-conceived pregnancies, Matorras et al. (1) identified 1,582 ectopic pregnancies, including 32 cases of cervical pregnancy, corresponding to an incidence of 3.5 per 10,000 pregnancies. The pathogenesis of cervical pregnancy remains unclear. Several potential contributing factors have been proposed. Patients undergoing ART may have endometrial abnormalities that impair embryo implantation and increase the risk of miscarriage. Individuals with a history of induced abortion and uterine curettage may experience additional endometrial damage, further reducing implantation potential. Moreover, dilation and curettage (D&C) procedures can injure the cervical canal, permitting endometrial tissue implantation at that site. Such changes can alter cervical vascularity and histology, thereby facilitating embryonic implantation within the cervical canal and leading to cervical pregnancy (1). Other studies suggest that rapid transport of the fertilized ovum or poor endometrial receptivity may allow the embryo to traverse the uterine cavity and implant within the cervical region. Additionally, some researchers have proposed that fertilization may occasionally occur directly within the cervical canal, followed by local implantation (2).

## Case presentation

### Case description

A 38-year-old woman was admitted with the chief complaint of amenorrhea for more than 2 months accompanied by vaginal bleeding for 18 days. Her menstrual cycles had previously been regular, with normal flow and color and only occasional dysmenorrhea. The last menstrual period occurred on 12 May 2025, with normal flow and duration. Forty-five days later, she developed persistent vaginal bleeding of variable intensity, at times exceeding her usual menstrual flow. She reported intermittent lower abdominal discomfort without significant pain and did not initially seek medical attention. On the 63rd day of amenorrhea, the patient developed nausea and vomiting. Ultrasonography performed at our hospital revealed a gestational sac located within the cervical canal, suggesting a possible cervical pregnancy. Her serum human chorionic gonadotropin (hCG) level was 46,879 mIU/ml. Based on these findings, a diagnosis of suspected cervical pregnancy was made, and the patient was admitted for further evaluation and management. The patient's past medical history was unremarkable. She had no chronic diseases, no history of surgery or trauma, and no known food or drug allergies. Her obstetric history included two prior induced abortions and two full-term vaginal deliveries (in 2018 and 2020). Physical examination revealed a distended, barrel-shaped cervix with a dilated external os and thin cervical lips. An irregular, decidual-like tissue measuring approximately 2 × 1 cm was visible within the cervical canal, with no evidence of active bleeding.

### Diagnostic assessment

**Figure 1:**
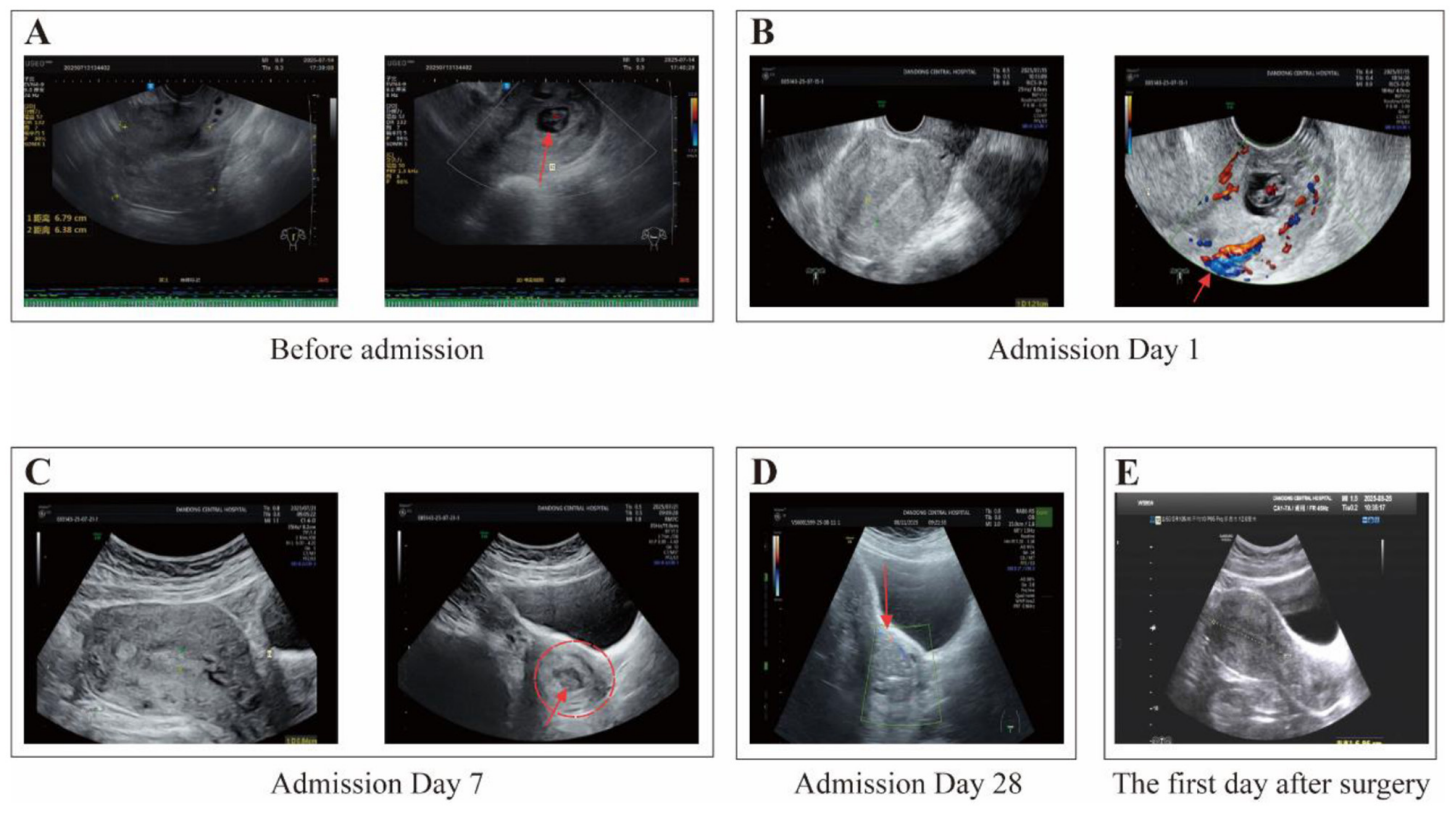
Serial TVUS of a cervical ectopic pregnancy managed with MTX plus adjunct TCM, followed by suction curettage. **(A)** Baseline (pre-admission): Grayscale TVUS shows a gestational sac implanted within the endocervical canal (→). **(B)** Hospital day 1: color Doppler confirms cervical implantation and reveals marked peritrophoblastic vascularity at the implantation site (→). **(C)** Hospital day 7 (after first MTX dose): Grayscale TVUS shows a non-viable pregnancy (○); the embryonic pole (→) exhibits no fetal cardiac activity, consistent with embryonic demise. **(D)** Hospital day 28 (after combined medical therapy, preoperation): Grayscale and color Doppler imaging reveal a heterogeneous echogenic mass within the cervical canal, measuring 4.0 × 3.3 × 3.0 cm by calipers. The previously prominent peritrophoblastic flow has regressed to scant focal signals (→), indicating substantial devascularization. **(E)** Postoperative day 5: follow-up grayscale TVUS confirms complete resolution of the cervical lesion with restoration of normal cervical and uterine anatomy. Interpretation: The series documents progressive devascularization and degeneration of trophoblastic tissue under medical therapy, facilitating safe definitive surgical evacuation. TVUS, transvaginal ultrasonography; MTX, methotrexate; TCM, traditional Chinese medicine.

**Figure 2:**
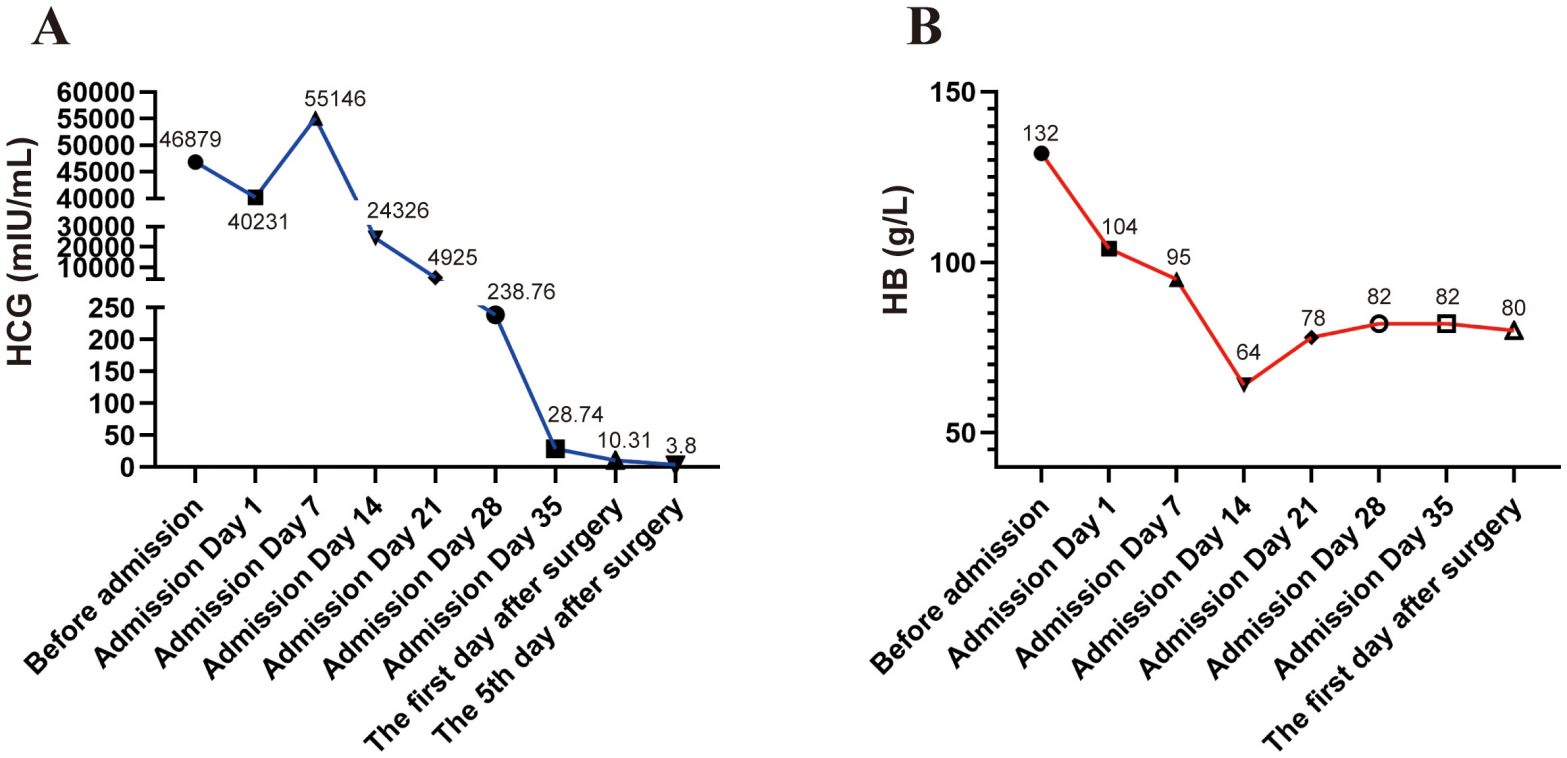
Line chart of changes in hCG and Hb during the treatment of cervical ectopic pregnancy. **(A)** Serum hCG trajectory from the pre-admission baseline to the postoperative follow-up. Concentrations peaked on hospital day 7 (54,326 mIU/ml) and then declined rapidly after the initiation of medical therapy and subsequent surgical evacuation, consistent with an effective treatment response. The reference range for non-pregnant women was hCG <5 mIU/ml. **(B)** Hb concentrations are shown over the same interval. Levels fell from admission and reached a nadir on hospital day 14, consistent with acute hemorrhage characteristic of cervical pregnancy. Values then increased and stabilized with combined medical, supportive, and surgical management. Anemia grading (WHO) was as follows: normal ≥120 g/L; mild 110**–**119 g/L; moderate 80**–**109 g/L; and severe <80 g/L. hCG, human chorionic gonadotropin; Hb, hemoglobin; WHO, World Health Organization. Units: hCG in mIU/ml; Hb in g/L.

The primary clinical manifestation of cervical pregnancy is painless vaginal bleeding, which typically progresses from light to heavy flow, though it may also present as intermittent episodes of heavy bleeding. In the present case, the patient mainly experienced intermittent vaginal bleeding; however, previous reports have described abdominal pain as the predominant symptom in some patients (3). The diagnosis of cervical pregnancy is primarily based on ultrasonographic findings. Diagnostic criteria include dilation of the cervical canal, visualization of a gestational sac or placental tissue within the canal (often accompanied by fetal cardiac activity or detectable blood flow), absence of intrauterine gestational structures, and the presence of an intact endometrial line. Cervical pregnancy is frequently misdiagnosed as inevitable abortion. Serial ultrasound examinations are useful for establishing a definitive diagnosis by demonstrating persistent characteristic cervical changes. In this case, the combination of serial ultrasonography (Figures 1A–C) and quantitative hCG monitoring (Figure 2A) confirmed the diagnosis of cervical pregnancy rather than threatened miscarriage.

### Therapeutic intervention

Upon admission, the patient experienced a transient episode of increased vaginal bleeding, estimated at approximately 200 ml. She declined interventional management and was instead treated symptomatically with fluid replacement and hemostatic therapy, after which the bleeding subsided. A follow-up ultrasound performed 1 day after admission confirmed the persistence of a cervical gestational sac containing a viable embryo. Physical examination showed cervical dilation with thinned margins and necrotic tissue visible within the cervical canal. Bimanual examination revealed a slightly enlarged uterine body, a thickened cervical canal, and a gourd-shaped uterus. Serum hCG levels were elevated. Vital signs were stable, and there were no contraindications to medical therapy. The patient received an intramuscular dose of methotrexate (MTX) at 50 mg/m^2^. The patient underwent weekly laboratory monitoring throughout the treatment course, which included complete blood count (CBC), liver and renal function tests, and serum hCG levels. The CBC was essential for detecting potential infection, monitoring for worsening anemia, and assessing hematological adverse effects associated with methotrexate. Liver and renal function tests were performed to evaluate for potential drug-induced toxicity and to identify any contraindications for continued treatment. Serial measurements of serum hCG levels served as the primary indicator for evaluating therapeutic response (Supplementary Table S1). On day 7 of hospitalization, hCG levels had increased, and ultrasound continued to show a cervical pregnancy without detectable fetal cardiac activity. As the treatment response was considered favorable, a second course of MTX was administered, combined with an adjuvant regimen of traditional Chinese medicine (TCM) composed of red peony root, Sargentodoxa cuneata, boiled and peeled peach seed, safflower, dried ginger, danshen root, Sparganii (vinegar-fried), zedoary (vinegar-simmered), Viola philippica, Aurantii Fructus Immaturus (fried bran), black-leaved honeysuckle, and centipede. The dosage and effects of Chinese herbal formulas are detailed in Table 1. Each dose of medication should be decocted into 200 ml of liquid and taken orally in two separate doses. After taking oral Chinese herbal medicine for 4 days, the patient experienced slight vaginal bleeding. We replaced dried ginger with largehead atractylodes rh in the original formula and added 10 g of fennel (roasted in salt water), 15 g of glossy privet fruit (stewed in wine), and 20 g of weeping forsythia capsule. On day 14, the patient experienced transient heavy vaginal bleeding estimated at approximately 400 ml. She also underwent a brief syncope episode and exhibited symptoms of anemia, including dizziness and fatigue. A subsequent gynecological examination revealed small clots within the vagina but no active bleeding. The cervical contour remained unchanged, and a gestational sac measuring approximately 3 × 2 centimeter (cm) was visible at the external os. Laboratory tests revealed serum hCG levels decreased by 50% compared to day 7, indicating a favorable decline in hCG. However, hemoglobin levels showed a significant decrease. Following an 800 ml packed red blood cell transfusion, the patient's hemoglobin levels improved. Liver function tests (transaminase levels) showed no abnormal elevation. A third course of MTX was then administered, during which no significant vaginal bleeding occurred. On day 21, hCG levels had markedly declined (Figure 2A), hemoglobin remained stable without further decrease (Figure 2B), and transaminase levels remained within normal limits. A fourth course of MTX was administered. On day 28, hCG continued to decline, and ultrasonography revealed no identifiable gestational sac. However, a 4.0 × 3.3 × 3.0-cm mixed-echo mass with detectable blood flow signals was observed in the cervical canal (Figure 1D). Transaminase levels remained stable, and supportive TCM therapy was continued. Based on the favorable therapeutic response, characterized by a progressive decline in serum beta-human chorionic gonadotropin (β-hCG) levels and a reduction in the cervical mass size, the Chinese herbal formula was modified (Supplementary Table S2). The formulation was adjusted to a granule preparation for enhanced patient compliance. These granules are produced from single-herb decoctions through a standardized pharmaceutical process that includes water extraction, separation, concentration, drying, and granulation. For administration, the contents of all sachets for one daily dose were dissolved together in 200 ml of boiling water. The resulting solution was stirred thoroughly to ensure complete homogenization and administered in two divided doses. On day 35, hCG levels showed a further significant decrease, and hemoglobin levels had slightly improved. Ultrasound revealed a reduction in the size of the cervical mixed-echo lesion with no significant blood flow signals. Ultrasound-guided evacuation of products of conception from the cervical canal was subsequently performed. Intraoperative blood loss was minimal.

**Table 1:** Composition and main efficacy of herbal formula.

**No**.	**Latin name**	**English name**	**Pinyin**	**Dose**	**Primary actions**
1	*Paeoniae Radix Rubra*	Red peony root	Chì Sháo	15 g	Clears heat and cools the blood, dispels stasis, and relieves pain. Indicated for amenorrhea, dysmenorrhea, and abdominal pain due to masses
2	*Sargentodoxae Caulis*	Sargentodoxa	Ji Xuè Téng	15 g	Promotes blood circulation and nourishes blood; regulates menstruation and relieves pain. Indicated for irregular menstruation, dysmenorrhea, amenorrhea, and blood deficiency with pallor
3	*Persicae Semen* (processed)	Peach seed	Chǎo Táo Rén	15 g	Promotes blood circulation and removes blood stasis. Indicated for amenorrhea, dysmenorrhea, masses, and abdominal distension
4	*Carthami Flos*	Safflower	Hóng Huā	15 g	Promotes blood circulation and menstruation, dispels blood stasis, and relieves pain. Indicated for amenorrhea, dysmenorrhea, retained lochia, masses and lumps, and abdominal pain due to blood stasis
5	*Zingiberis Rhizoma*	Dried ginger	Gān Jiāng	10 g	Warms the middle and dispels cold, restores yang, and unblocks the meridians. Indicated for cold pain in the epigastrium and abdomen
6	*Salviae Miltiorrhizae Radix et Rhizoma*	Danshen root	Dān Shēn	15 g	Promotes blood circulation and removes blood stasis; regulates menstruation and alleviates pain; calms the mind and relieves restlessness; cools the blood and dissipates abscesses. Indicated for masses and accumulations, heat-induced arthralgia and pain, restlessness and insomnia, irregular menstruation, dysmenorrhea, and amenorrhea
7	*Sparganii Rhizoma* (vinegar-fried)	Sparganii	Cù Sān Léng	15 g	Promotes blood circulation and qi flow, eliminates stagnation, and relieves pain. Indicated for masses and lumps, dysmenorrhea, and amenorrhea due to blood stasis
8	*Curcumae Rhizoma* (vinegar-simmered)	Zedoary	Cù É Zhú	15 g	Promotes qi circulation and breaks up blood stasis; eliminates accumulations and relieves pain. Indicated for masses and nodules and menstrual disorders due to blood stasis
9	*Violae Herba*	Viola philippica	Zi Huā Dì Ding	25 g	Clears heat and detoxifies, cools the blood, and reduces swelling
10	*Aurantii Fructus Immaturus* (bran-fried)	Aurantii Fructus Immaturus	Fǔ Chǎo Zhi Shí	15 g	Dispels stagnation and eliminates accumulation; transforms phlegm and disperses fullness. Indicated for internal accumulation and stagnation
11	*Ecliptae Herba*	Black-leaved honeysuckle	Mò Hàn Lián	25 g	Nourishes the liver and kidneys, cools the blood, and stops bleeding. Indicated for yin deficiency with blood heat, metrorrhagia, and uterine bleeding
12	*Scolopendra*	Centipede	Wú Gong	4 pcs	Calms wind and relieves spasms, unblocks meridians, and alleviates pain

The above drug information is sourced from the 2025 edition of the Pharmacopeia of the People's Republic of China.

Latin scientific name: The botanical name of this herb, with genus and species names indicated in italics.

English name: The common English name of this herb.

Pinyin: The standardized Romanized spelling of the names of Chinese herbal medicines.

Dose: Typical dose used in the formula (g = g; Pcs = pieces).

Main functions: The main pharmacological effects and clinical indications of this herb.

Abbreviations and symbols: g, grams; pcs, pieces.

Processing, vinegar stir-frying, bran stir-frying, and vinegar roasting: Refer to specific processing methods applied to medicinal herbs to alter their medicinal properties or enhance their therapeutic effects.

### Follow-up and outcomes

**Figure 3:**
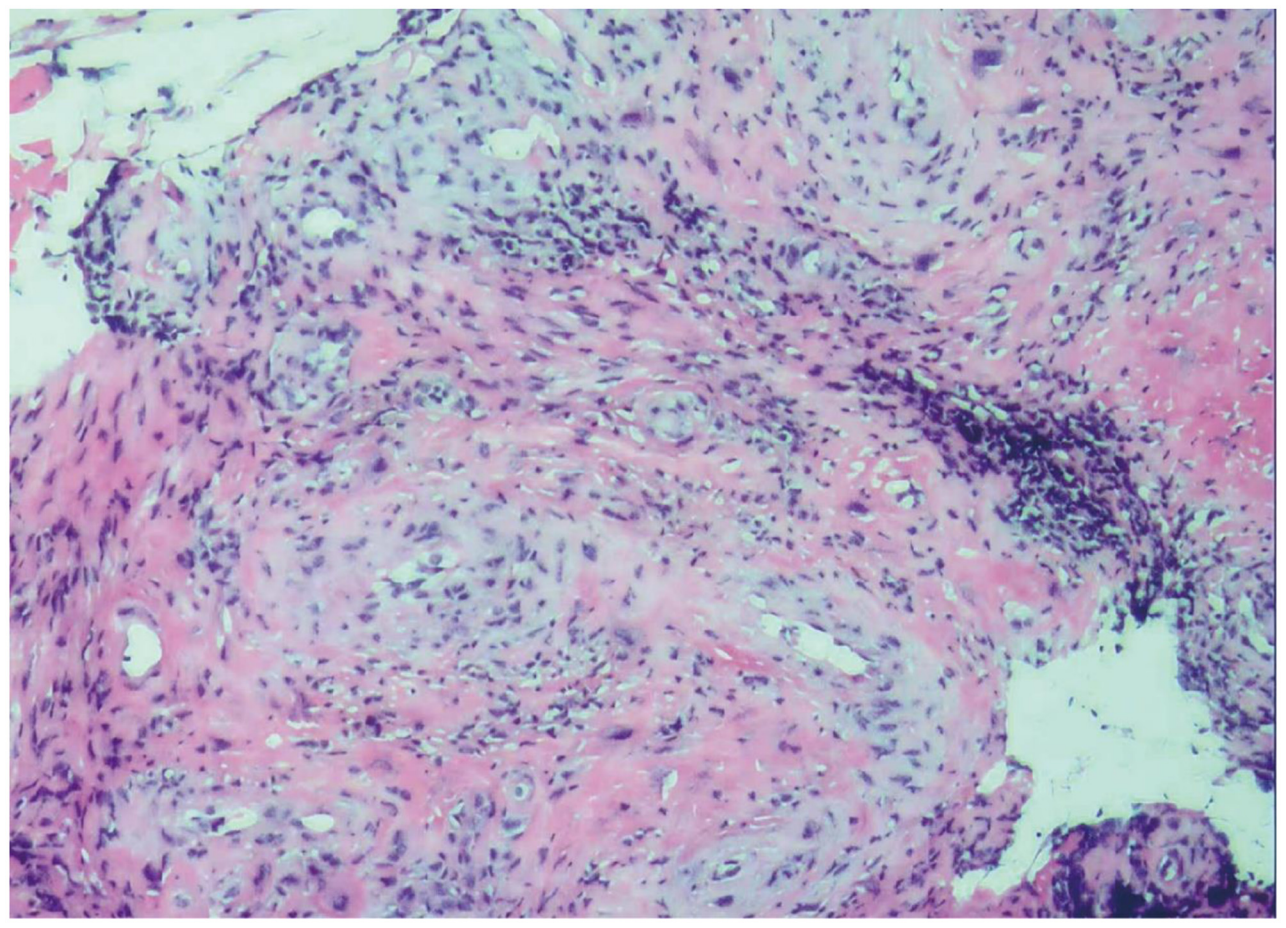
Pathological examination results of postoperative cervical canal scraping material. Under the microscope, villous tissue and nourishing leaf cells can be seen, which ultimately confirms the diagnosis of cervical pregnancy at the histological level.

One day postoperatively, hCG levels had decreased by approximately 50% compared to preoperative values. Five days after surgery, hCG levels became negative (Figure 2A), ultrasound findings were unremarkable (Figure 1E), and vaginal bleeding had resolved. Postoperative histopathological examination revealed degenerative decidual tissue and chorionic villi, along with a small amount of proliferative endometrium and cervical canal tissue. These findings were consistent with pregnancy (Figure 3). The patient's menstruation resumed 18 days after the procedure. As she expressed no current desire for future pregnancy, no fertility-related follow-up was pursued.

## Discussion

Through multiple courses of MTX monotherapy in combination with traditional Chinese medicine, a significant reduction in serum hCG levels was achieved. Following the decline in hCG, a uterine curettage was performed, successfully avoiding the need for a hysterectomy that might have been required due to excessive intraoperative bleeding. Preoperative examination found that the cervical morphology returned to normal, hCG turned negative in a short time after the operation, no cervical canal adhesion occurred, and menstruation was normal after the operation. However, the length of hospital stay was long, and two instances of transient vaginal bleeding occurred during the treatment, and hemoglobin decreased, requiring blood transfusion.

The cervix is composed mainly of connective tissue, whereas the uterine body primarily consists of smooth muscle. Owing to these structural distinctions, hemorrhage occurring in the cervical region is difficult to control effectively with conventional uterotonic agents (4). Historically, due to the limitations of medical technology, hysterectomy was the predominant treatment for cervical pregnancy. Although this approach effectively mitigated the risk of hemorrhagic complications, it frequently resulted in the irreversible loss of fertility. With ongoing advances in medical science, therapeutic strategies for cervical pregnancy have become increasingly diverse; however, a standardized treatment protocol has yet to be established. Paola Bianchi et al. proposed that the management of cervical pregnancy should be guided by three key principles: (1) minimizing the risk of hemorrhage, (2) removing all gestational tissue, and (3) preserving reproductive function (5).

For patients with cervical pregnancy, early and accurate diagnosis, along with prompt and effective treatment, is essential. Therapeutic strategies should be individualized according to the patient's clinical profile. Current management options encompass both surgical and conservative approaches, with commonly used protocols including systemic or local administration of MTX, local potassium chloride injection, hysteroscopic removal, and uterine artery embolization (UAE). Selection of the optimal treatment should take into account factors such as gestational age, serum β-hCG levels, the presence and severity of active bleeding, fertility preservation desire, concomitant intrauterine pregnancy, and the experience of the attending clinician. Dr. Aya Mohr-Sasson successfully performed a one-step hysteroscopic removal of gestational tissue in a patient diagnosed with a cervical pregnancy. She concluded that hysteroscopic surgery may be safe when cases are appropriately selected, but factors such as gestational age, HCG levels, and embryo viability may influence the procedure's success rate, warranting further research (3).

Literature reports indicate that ultrasound-guided intracystic injection therapy for patients with cervical pregnancy can be performed via either transabdominal or transvaginal routes under local anesthesia. Clinical studies have demonstrated a high success rate (94%) for ultrasound-guided local MTX injection combined with systemic therapy, compared with 81% for systemic MTX alone (6). M. Yamaguchi et al. further confirmed that a single ultrasound-guided local MTX injection is effective for preserving fertility, although no standardized protocol currently exists for local injection dosing. Szmygin et al. (7) reported that treatment strategies for cervical pregnancy may also involve uterine artery embolization (UAE) combined with MTX administration and curettage. In their study, nine patients underwent bilateral UAE. Follow-up assessments showed the return of menstruation within 30 days in six patients and within 3 months in the remaining three (7). However, the use of UAE remains controversial, particularly regarding the risk of delayed hemorrhage. Reported cases indicate that late bleeding can be effectively managed using a gelatin hemostatic matrix composed of human thrombin and bovine gelatin, which achieves hemostasis by exerting pressure through expansion (8). If gestational tissue is implanted deeply within the cervical muscular layer and cannot be completely removed by curettage, collateral circulation and recanalization of the uterine arteries may allow residual tissue to persist and proliferate. Whether UAE induces permanent ovarian failure in patients with pre-existing diminished ovarian reserve remains to be elucidated. Thus, the potential impact on fertility and ovarian reserve must be carefully considered when selecting this treatment modality. Alternative therapeutic strategies have also been explored, including double-balloon catheter therapy for cervical pregnancy and cervical ripening with induction catheters to terminate fetal cardiac activity and prevent hemorrhage. While these approaches avoid more invasive procedures, their widespread clinical adoption requires further validation in larger patient cohorts (9).

Additionally, local injection of absolute ethanol has been utilized as an alternative therapeutic approach for the management of cervical pregnancy. This method has been shown to induce a significant decline in serum β-hCG levels within 2 h after the initial injection, effectively and rapidly terminating trophoblastic cell activity. If the first administration fails to achieve the desired effect, absolute ethanol can be safely re-administered without causing clinically significant impairment of hematopoietic function (10).

The integrated Chinese and Western medicine approach has demonstrated favorable clinical efficacy as a conservative treatment strategy for cervical pregnancy. Its typical treatment regimen often combines MTX, mifepristone, and traditional Chinese medicine formulas that promote blood circulation and resolve stasis. Within the theoretical framework of traditional Chinese medicine, cervical pregnancy falls under the syndrome pattern of “blood stasis in the lower abdomen, pain due to obstruction.” Its core pathogenesis lies in blood stasis obstruction and meridian blockage. Addressing this pathogenesis, TCM treatment follows the principles of “promoting blood circulation and resolving stasis, dispersing nodules and eliminating masses, and unblocking meridians and activating collaterals.” This aims to facilitate the clearance and resorption of ectopic pregnancy tissue. Specifically, “promoting blood circulation” refers to enhancing the flow of qi (qi, a core concept in traditional Chinese medicine theory that describes vital energy and functional states) and blood to improve systemic and local circulation, while “resolving stasis” means dissipating stagnation to alleviate associated clinical symptoms (11).

Multiple herbal components in the formula exert therapeutic effects through synergistic mechanisms. For instance, Curcuma and Carthamus possess anti-implantation and abortion-inducing properties; the amygdalin in *Prunus persica* seeds hydrolyzes to produce hydrogen cyanide, which paralyzes the embryonic central nervous system and inhibits its development (12). *Salvia miltiorrhiza* and *Paeonia lactiflora* are the core herbs responsible for promoting blood circulation and resolving stasis. Research confirms that the primary active components of *Salvia miltiorrhiza* (such as phenolic acids and tannic acids) exert vascular endothelial protection by reducing oxidative stress and inflammatory damage and regulating the nitric oxide/endothelin-1 (NO/ET-1) balance. Additionally, *Salvia miltiorrhiza* enhances anticoagulant and fibrinolytic activity, inhibits platelet activation and aggregation, and possesses vasodilatory effects. Its lipid-regulating and blood rheology-improving properties are also considered potential mechanisms for its anti-blood stasis effects (13). Regarding analgesia, animal studies indicate that paeonol in red peony root alleviates pain by modulating type 1 and type 2 polarization of microglia to attenuate central sensitization (14). Simultaneously, both chrysanthemum (Li et al. (15) reported that it contains 135 chemical constituents, primarily terpenoids and flavonoids) (15) and salvia extracts (such as Salviplenoid A) in the formula have exhibited anti-inflammatory activity (16). These components may reduce the risk of cervical adhesions by alleviating local inflammatory responses.

MTX currently forms the foundation of medical treatment for cervical pregnancy, used either alone or in combination with other therapies. However, MTX therapy carries a risk of significant vaginal hemorrhage. The inclusion of *Eclipta prostrata* (documented in the Chinese Pharmacopeia for its hemostatic properties) in this formula may help counteract this bleeding tendency. Additionally, as a chemotherapeutic agent, MTX carries potential adverse effects such as bone marrow suppression and damage to liver and kidney function. From a traditional Chinese medicine perspective, MTX exhibits a cold nature. The warming and cold-dispelling properties of dried ginger in the formula can partially counteract this coldness. Furthermore, phloretin (PHI), a natural lignan derived from forsythia, has been shown by Ji et al. to significantly suppress systemic inflammatory responses through potent anti-inflammatory effects (17). This finding indicates that forsythia's heat-clearing and toxin-resolving properties may help mitigate some of the toxic side effects induced by MTX.

The patient in this case received combined treatment with MTX and traditional Chinese medicine. Although no liver or kidney dysfunction occurred during the treatment, transient heavy vaginal bleeding was observed, accompanied by a decrease in hemoglobin levels requiring blood transfusion intervention. This was presumed to be related to a rapid decline in serum β-hCG levels. Following four MTX administrations, the patient continued monotherapy with traditional Chinese medicine. During this phase, β-hCG levels continued to decline, confirming the efficacy of the combined treatment regimen while highlighting the personalized advantage of TCM therapy, which can be adjusted according to disease progression. Caution is warranted, as TCM herbs promoting blood circulation and resolving stasis may exacerbate bleeding risks during active hemorrhage. In such cases, dosage reduction or temporary discontinuation should be considered, accompanied by enhanced clinical monitoring. Throughout the entire treatment process, an emergency surgery plan must be readily available to address potential treatment failure or life-threatening hemorrhage.

Currently, the combined use of TCM and MTX for treating cervical pregnancy still faces numerous challenges. These primarily include insufficient quantitative assessment of synergistic effects between Chinese and Western medicine, as well as incomplete elucidation of the molecular mechanisms underlying TCM activity. Consequently, clinical dosages and treatment durations largely rely on physician experience, lacking standardized guidelines based on high-level evidence. Future research urgently requires more rigorously designed clinical trials and in-depth exploration of underlying mechanisms to optimize treatment protocols and provide robust scientific evidence for the widespread adoption of this combined therapeutic strategy.

## Conclusion

In this case of cervical pregnancy, the combination of MTX and TCM was associated with successful conservative management, culminating in complete biochemical and clinical recovery without hysterectomy. The treatment course, marked by rapid β-hCG decline and no observed hepatic or renal impairment, provides preliminary clinical evidence that supports the hypothesis of TCM augmenting MTX's effects while reducing its toxicity. It is crucial to interpret these results as hypothesis-generating rather than conclusive; they indicate a potential but do not prove efficacy. This report underscores the need for prospective, controlled investigations to validate whether this integrative strategy can consistently improve outcomes and expand the scope of fertility-sparing interventions.

## Patient perspective

She felt reassured by the doctor's patient explanation and treatment when she sought medical attention for vaginal bleeding after her period stopped and was diagnosed with a cervical pregnancy. She stated that although bleeding occurred during the treatment, her condition was ultimately controlled without requiring a hysterectomy, and she expressed deep gratitude to the medical staff.

## Data Availability

The original contributions presented in the study are included in the article/Supplementary material, further inquiries can be directed to the corresponding author.
