# Supplementary material for: Cervical pregnancy treated with methotrexate combined with traditional Chinese medicine: a case report and literature review
Source: Front Med (Lausanne). 2025 Dec 5;12:1723052. doi: 10.3389/fmed.2025.1723052 (PMC12714626; doi:10.3389/fmed.2025.1723052)
Supplement: Supplementary file 1 [file Supplementary_file_1.docx]

**Supplementary Table 1. Serial laboratory parameters during medical management and the perioperative course of cervical ectopic pregnancy treated with MTX plus adjunct TCM.**

|  | **Before Admission** | **Admission Day 1** | **Admission Day 7** | **Admission Day 14** | **Admission Day 21** | **Admission Day 28** | **Admission Day 35** | **First Day After Surgery** |
| --- | --- | --- | --- | --- | --- | --- | --- | --- |
| **Hepatic Function** | | | | | | | | |
| AST (U/L) | - | 20 | 19 | 23 | 18 | 22 | 24 | 18 |
| ALT (U/L) | - | 21 | 23 | 25 | 36 | 31 | 28 | 22 |
| **Renal Function** | | | | | | | | |
| UREA (mmol/L) | - | 3.3 | 3.5 | 1.8 | 6.6 | 4.4 | 4.8 | 4.7 |
| CREA (μmol/L) | - | 38 | 40 | 41 | 37 | 37 | 41 | 39 |
| **Hematological Parameters** | | | | | | | | |
| WBC (×10⁹/L) | 8.59 | 6.98 | 8.23 | 12.61 | 8.04 | 6.39 | 6.92 | 8.44 |
| NE (×10⁹/L) | 5.80 | 4.54 | 5.93 | 7.95 | 5.95 | 4.03 | 5.01 | 6.00 |
| PLT (×10⁹/L) | 177 | 160 | 200 | 291 | 233 | 285 | 324 | 337 |

**Values were obtained weekly from pre-treatment baseline through postoperative day (POD) 1. Monitoring was designed to (1) evaluate potential MTX-related hepatotoxicity and nephrotoxicity and (2) characterize hematologic responses over time.**

**Summary of findings:**

**Hepatic enzymes (AST, ALT): Remained within reference intervals throughout, with no biochemical evidence of MTX-induced liver injury.**

**Renal indices (urea nitrogen, creatinine): Stable and within reference limits, consistent with preserved renal function and expected MTX elimination.**

**Hematologic profile: White blood cell count (WBC) and absolute neutrophil count (NE) fluctuated without sustained leukopenia or neutropenia, suggesting minimal myelosuppression at the administered dose. Platelet counts (PLT) rose pre-operatively, compatible with reactive thrombocytosis in the setting of inflammation and/or bleeding typical of cervical pregnancy; values stabilized after definitive management.**

**Abbreviations: AST, aspartate aminotransferase; ALT, alanine aminotransferase; UREA, urea nitrogen (blood urea nitrogen); CREA, creatinine; WBC, white blood cell count; NE, absolute neutrophil count; PLT, platelet count; MTX, methotrexate; TCM, traditional Chinese medicine; POD, postoperative day.**

**Supplementary Table 2. Composition of Ready-to-Use Granules of Traditional Chinese Medicine.**

| No. | Latin Name | English Name | Pinyin | Amount per Dose | Primary Actions |
| --- | --- | --- | --- | --- | --- |
| 1 | *Angelicae Sinensis Radix* | Chinese Angelica | Dāng Guī | 1 bag | Nourishes blood and promotes blood circulation; regulates menstruation and relieves pain. Indicated for blood deficiency with pallor and jaundice, dizziness and palpitations, irregular menstruation, amenorrhea and dysmenorrhea, and abdominal pain due to deficiency-cold. |
| 2 | *Paeoniae Radix Rubra* | Red Peony Root | Chì Sháo | 1 bag | Clears heat and cools the blood, dispels stasis and relieves pain. Indicated for liver qi stagnation with costal pain, amenorrhea with dysmenorrhea, and masses with abdominal pain. |
| 3 | *Astragali Radix* | Milkvetch Root | Huáng Qí | 1 bag | Tonifies qi and elevates yang, generates fluids and nourishes blood, dispels stagnation and unblocks obstruction, expels toxins and drains pus. Indicated for qi deficiency with fatigue, bloody stools and uterine bleeding, blood deficiency with pallor and jaundice. |
| 4 | *Codonopsis Radix* | Tangshen | Dǎng Shēn | 1 bag | Strengthens the spleen and nourishes the lungs; enriches blood and generates fluids. Indicated for spleen-lung qi deficiency, deficiency of qi and blood, sallow complexion, palpitations, and shortness of breath. |
| 5 | *Persicae Semen*(processed) | Peach Seed | Chǎn Táo Rén | 1 bag | Promotes blood circulation and removes blood stasis. Indicated for amenorrhea, dysmenorrhea, masses, and abdominal distension. |
| 6 | *Carthami Flos* | Safflower | Hóng Huā | 1 bag | Promotes blood circulation and menstruation, dispels blood stasis and relieves pain. Indicated for amenorrhea, dysmenorrhea, retained lochia, masses and lumps, and abdominal pain due to blood stasis. |
| 7 | *Salviae Miltiorrhizae Radix et Rhizoma* | Dan-Shen Root | Dān Shēn | 1 bag | Promotes blood circulation and removes blood stasis; regulates menstruation and alleviates pain; calms the mind and relieves restlessness; cools the blood and dissipates abscesses. Indicated for masses and accumulations, heat-induced arthralgia and pain, restlessness and insomnia, irregular menstruation, dysmenorrhea, and amenorrhea. |
| 8 | *Eupolyphaga seu Steleophaga* | Ground Beetle | Tǔ Biē Chóng | 2 bags | Breaks up blood stasis and expels stagnant blood. Indicated for amenorrhea due to blood stasis and postpartum abdominal pain caused by blood stasis obstruction. |
| 9 | *Sargentodoxae Caulis* | Sargentodoxa Stem | Dà Xuè Téng | 1 bag | Clears heat and detoxifies, promotes blood circulation, dispels wind and relieves pain. Indicated for amenorrhea and dysmenorrhea. |
| 10 | *Cyperi Rhizoma*  (vinegar-fried) | Nutgrass Galingale Rhizome | Cù Xiāng Fù | 1 bag | Soothes the liver and relieves depression, regulates qi and eases the middle burner, regulates menstruation and alleviates pain. Indicated for liver qi stagnation with distension and pain, irregular menstruation, amenorrhea, and dysmenorrhea. |
| 11 | *Linderae Radix* | Lindera Root | Wū Yào | 1 bag | Promotes qi circulation and alleviates pain; warms the kidneys and dispels cold. Indicated for cold stagnation with qi stagnation, menstrual cold abdominal pain, etc. |
| 12 | *Corydalis Rhizoma*  (vinegar-fried) | Corydalis Yanhusuo | Cù Yán Hú Suǒ | 1 bag | Promotes blood circulation, regulates qi flow, and relieves pain. Indicated for amenorrhea, dysmenorrhea, and postpartum blood stasis. |
| 13 | *Chrysanthemi Indici Flos* | Wild Chrysanthemum Flower | Yě Jú Huā | 1 bag | Clear heat and detoxify, reduce fire and calm the liver. |

**Latin Name: The internationally recognized botanical name for herbs.**

**English Name: The commonly used English name for herbs.**

**Pinyin: The Chinese pinyin name for herbs.**

**Amount per Dose: Refers to the number of bags of granules used each time.**

**Primary Actions: Describes the main pharmacological effects and indications of herbs in traditional Chinese medicine theory.**

**The above drug efficacy information is sourced from the 2025 edition of the Pharmacopoeia of the People's Republic of China.**

**Granules without decoction are granular preparations made from single-ingredient Chinese herbal slices through water extraction, separation, concentration, drying, and granulation.**
